# Contrast Volume for Preoperative CT Angiography of the Aorta Based on Patient Heart Rate and Body Surface Area: A Pilot Study

**DOI:** 10.5334/jbsr.1714

**Published:** 2019-10-04

**Authors:** Dominic Raymakers, Adriana Dubbeldam, Walter Coudyzer, Hilde Bosmans, Geert Maleux

**Affiliations:** 1 University Hospitals Leuven, BE

**Keywords:** computed tomography, aorta, CT-angiography, contrast agents, aneurysm, dose reduction

## Abstract

**Purpose:**

To evaluate contrast agent dose reduction using an algorithm based on heart rate and body surface area.

**Methods:**

A prospective study with 55 patients undergoing aortic CT was performed. Contrast agent dose, enhancement and image quality between three study groups were compared. Group 1: a fixed, standard dose of 120 ml, group 2: contrast agent dose calculated based on heart rate and body surface area, group 3: additional dilution of 50% of the calculated dose based on heart rate and body surface area.

**Results:**

The mean contrast agent dose in group 2 was reduced by 15% (P < 0.01) with unchanged high visual scoring in comparison to group 1. In group 3, contrast agent dose reduction was 60% (P < 0.01); average image quality dropped 19% (P < 0.01) compared to group 1, but was still sufficient, except for two patients.

**Conclusions:**

Contrast agent dose was significantly reduced without compromising diagnostic efficacy using the proposed algorithm.

## Introduction

Computed tomography (CT) angiography of the aorta is considered to be a valuable tool for detection and follow-up of abnormalities of the thoraco-abdominal aorta, including aneurysmal dilatation, dissection, penetrating ulcer, inflammation of the aortic wall, luminal stenosis and embolic events.

In many centers a standardized scanning protocol for CT aortography with administration of a fixed dose of non-ionic contrast medium is used. A standard dose of 120 milliliter (ml) has been used for many years in our institution as well as many others. However, due to advances in CT technology and image reconstruction—including tube voltage, faster image acquisition and multiphasic contrast injection implementation—lower contrast doses have been proposed by some authors [1,2,3,4,5,6,7,8]. Other authors have achieved some level of contrast dose reduction by interactive injection of contrast, where flow of contrast is stopped when scanning is started [9].

Finally, it has been demonstrated [10,11,12,13,14] that contrast enhancement is highly dependent on patient habitus, and more in particular that contrast dose should be adjusted for body surface area (BSA), a measure calculated from both body weight and height factors. Two other important factors affecting contrast enhancement are cardiac output and cardiovascular circulation [15]. Contrast medium is diluted and cleared faster with higher cardiac output, but the latter is not easily available in all patients while heart rate can be measured with simple non-invasive devices. Therefore, a practical calculator was created to assess the optimal contrast dose based on patient heart rate and BSA.

The aim of this study is to assess whether a new contrast agent dose calculator could be used for CT angiography of the aorta. Appropriateness was judged from a quantitative and qualitative scoring of image quality, in a comparative study with a fixed contrast agent dose of 120 ml. In a second phase we have also assessed whether we could further dilute the calculated volumes by 50%. As we use a constant contrast injection rate, the 50% dilution increases the duration of the bolus.

## Materials and Methods

### Study design

A single-center prospective study of patients undergoing a CT-angiography of the thoraco-abdominal or abdominal aorta for the detection or follow-up of aortic aneurysm or dissection was performed. This study was approved by the local Ethics Committee (the protocol number of the ethics committee approval is S0215861). Patients gave informed consent and were allocated to one of three study groups and scanned following a standardized scanning-protocol, only changing contrast dose between groups. Dose calculation was performed by an injection calculator (iCalc, Medicor International, Herent, Belgium), which was incorporated in the contrast dose injector on-site, to allow easy input by the CT-technicians before scanning. The algorithm for dose calculation is summarized in paragraph.

### Contrast agent dose calculation

Three study groups were created: group 1 (control group) received a standard contrast dose of 120 ml; group 2 (calculator group) received an injector-calculated contrast agent dose (40–150 ml), and group 3 (calculator + dilution group) received an additional dilution of 50% on top of the injector-calculated dose.

In case of a mild to moderate degree of renal impairment (creatinine 1.5–2.0 mg/dl), patients were randomly assigned to group 2 or 3, to allow contrast dose reduction in these patients. Also in the absence of any contrast agent study protocol, contrast agent dose would have been reduced in these patients, typically by 20%.

### Patient enrollment

A total of 55 patients were included and randomized using a sealed envelope system to one of the three scan groups. A total of 60 CT scans was performed. Two patients had a second follow-up scan (at six months) within the study period and were enrolled in another patient group, to allow intra-patient comparability. Also, in three patients, a previous CT scan outside the study period was retrospectively collected to achieve intra-patient comparability. Criteria for these collected scans were an exact same scanning protocol (same CT scanner) and same contrast concentration with a standard contrast dose of 120 ml in the previous scan.

Patients in follow-up for cardiac disorders were excluded. Patients with contra-indications for contrast administration (significant adverse reactions or creatinine above 2.0 mg/dl), as well as patients with intravenous access, not allowing a bolus contrast injection of 4 cc/sec, were excluded from this study. In case of moderate renal impairment, patients were randomly assigned to group 2 or 3 to receive a reduced contrast dose.

CT-aortography was performed for diagnosis or follow-up of aneurysms, dissection or aortitis. Patients with previous aortic surgery including endovascular aortic repair (EVAR) or stenting were excluded. All CT measurements and assessments were performed in the abdominal aorta, including patients with abdominal and thoraco-abdominal CT-aortography. Thoracic CT-aortography was excluded from the study.

### Contrast agent dose calculation

A calculating device (iCalc, Medicor International, Herent, Belgium) was used to define the optimal contrast dose. The formula on which the calculator is based consists of four components. First, BSA is calculated using the following parameters: ((length (cm) × weight (kg))/3600) × 0.5. Secondly, BSA is multiplied by a fixed contrast dose of 45 ml/m². This was based on the findings of a Japanese group [14] showing that aortic enhancement tended to be consistent using a protocol in which a fixed value of 42.51 ml/m² was administered. We arbitrarily set our value to 45 ml/m². Thirdly, the contrast volume was adjusted for heart rate using fixed threshold values, summarized in Table 1. Finally, an adjustment was made according to the used iodine concentration, also summarized in Table 1.

**Table 1:** Contrast volume correction for heart rate and contrast medium concentration.

Heart rate (beats per minute)	Volume correction (ml)

<55	–10
56–65	+0
66–75	+10
76–90	+20
91–105	+25
>105	+30
Contrast medium concentration (mg I/ml)	
>350	–2 ml per 10 mg I/ml above 350
<350	+2 ml per 10 mg I/ml below 350

This information was put into the calculator by one of the scanning technicians, after which the calculator assessed the optimal contrast agent dose to be administered. After either adjustment or confirmation, contrast agent injection was performed at a standard rate of 4 cc/second and followed by a 30 ml 4cc/second saline flush.

### Scanning protocol

All acquisitions were performed on a 128-detector-row CT (Siemens Somatom Definition FLASH, Erlangen, Germany).

All patients were scanned in single-energy mode. The imaging parameters were detector collimation, 128 × 0.6 mm; helical pitch, 1.2; gantry rotation time, 0.5 second; tube voltage for normal size patient, 120 kV; and planned tube current–time product per rotation for a normal patient size, 210 mAs. Automatic tube current modulation adjusted the tube loads to different patient sizes.

Patients with an aneurysm received a native and arterial phase CT. Patients with a dissection received an arterial phase CT followed by a venous phase CT. Scan delay was individualized using a bolus-tracking technique. Injection rate was fixed at 4 cc/sec for all patients. Triggering was performed at intra-arterial enhancement of 120 HU at the diaphragmatic region.

In patients with thoraco-abdominal pathology, field of view included the clavicles to the bilateral inguinal region; in abdominal aortic pathology, field of view included diaphragms to the bilateral inguinal region. Reconstructed images of 3 mm and 1 mm were available in axial reformation, and coronal and reformation was made available in 3 mm thickness for viewing on the PACS system.

Contrast medium was either Iodixanol 320 (Visipaque, GE Healthcare, 320 mg I/ml), Iomeprol 350 (Iomeron, Bracco, 350 mg I/ml) or Iobitridol 350 (Xenetix, Guerbet, 350 mg I/ml). Stock contrast medium concentration was adjusted in the injector calculator and recorded in the data. Contrast dose was either 120 ml (standard dose, group 1), a calculated dose (limit 40 ml–150 ml), or a calculated dose with a 50 percent extra dose reduction (lower limit 20 ml).

### Quantitative image analysis

Quantitative assessment was performed by measurement of HU at fixed levels in the abdominal aorta, including the suprarenal level (coeliac trunk), renal level, infrarenal level (midaorta), and in both common iliac arteries. A circular region of interest (ROI) was carefully placed in the center of the aorta or, in case of dissection, central in the true lumen. For comparison purposes, a mean for the five levels together was assessed for every patient. Finally, the lowest and highest values at any level were also analyzed for comparison.

### Qualitative image analysis

Two experienced vascular radiologists using visual grading analysis performed qualitative assessment. Readings were done independently. Also, a second reading was performed by one of the vascular radiologists more than one month later, to allow intra-observer comparison.

Visual scoring was based on a five point visual scale (1 = inadequate/non-diagnostic; 2 = suboptimal; 3 = sufficient/diagnostic; 4 = good; 5 = excellent). A clinical score of three or more was considered clinically acceptable, whereas a score of one was inacceptable for diagnostic purposes.

### Radiation dose collection

For all patients the kilo-voltage (kV), milli-Amperes (mAs), the volume CT dose index (CTDI vol) and dose-length product (DLP) were collected for the pre-contrast as well as the arterial phase CT scan. Dose comparison was performed for the arterial phase.

### Statistical analysis

Patient parameters (age, weight, height, body mass index and heart rate) are presented as mean, standard deviation and range (minimum–maximum). In case of continuous variables (contrast dose, enhancement, radiation dose), statistics were performed by using a paired two-tailed Student’s t-test and presented as mean, standard deviation, median and range. Ordinal variables (visual grading) were presented as median and range. Inter- and intra-observer correlation was evaluated by intraclass correlation coefficient (ICC). Differences were considered to be statistically significant at *P* < 0.025 (Bonferroni-correction α/2).

## Results

60 CT-angiography scans were performed in a total of 55 patients (16 female, 39 male; median age 66 years; range 22–87 years). In three patients, scanned in either group 2 or 3, a previous CT scan meeting the study criteria (120 ml contrast dose of 350 mg/ml concentration) was available and subsequently collected. Two patients scanned in group 1 received a follow-up CT-scan within the study period and were scanned in group 2 (one patient) or group 3 (one patient) for comparison.

In 15 of 60 CT scans the scanned region included the abdomen, in the remaining 45 CT scans both the thorax and abdomen were scanned. Thirty scans were performed for the detection or follow-up of aortic aneurysm. One patient was scanned for suspected aortitis, and 29 scans were performed for the detection or follow-up of aortic dissection. Patient characteristics are summarized in Table 2.

**Table 2:** Patient demographics.

	Group 1	Group 2	Group 3

Age (years)	65.8 ± 16.8 (67, 22–87)	66.6 ± 8.7 (68, 52–82)	64.1 ± 14.3 (68, 25–84)
Sex (male/female)	11/9	15/5	15/5
Weight (kg)	74.6 ± 15.0 (75.5, 50–105)	82.6 ± 14.0 (82.5, 50–115)	79.9 ± 14.3 (81.5, 60–109)
Height (cm)	167.9 ± 10.0 (168, 150–186)	172.7 ± 8.2 (172, 154–187)	172.1 ± 11.0 (174.5, 152–186)
Body mass index (kg/m^2^)	26.3 ± 3.8 (26, 20.8–33.9)	27.5 ± 3.2 (28.3, 21.1–33.6)	26.8 ± 2.8 (27.8, 21.1–31.8)
Heart rate (bpm)	66.8 ± 15.6 (68, 39–95)	72.9 ± 18.4 (67, 51–122)	66.7 ± 18.5 (60, 49–127)
Th – Abd/Abd	17/3	15/5	13/7
Aneurysm/dissection/other	9/10/1	11/9/0	10/10/0

Data presented as mean ± standard deviation (median, minimum-maximum).

### Contrast agent dose calculation

Contrast doses for the three study groups are summarized in Table 3.

**Table 3:** Summary of results.

	Group 1	Group 2	Group 3	Difference and p-value		

				Group 1 vs 2	Group 1 vs 3	Group 2 vs 3

Contrast dose (ml)	120 (120–120)	101.8 ± 23.2 (102.5, 42–150)	48.1 ± 12.2 (46, 29–72)	–15% (p < 0.01)	–60% (p < 0.01)	–53% (p < 0.01)
Average enhancement (HU)	282.2 ± 82.1 (284.5, 156.3–569.8)	279.3 ± 68.7 (284.5, 155.6–421.3)	191.2 ± 79.3 (191.5, 79.1–449.1)	–1% (p 0.91)	–32.2% (p < 0.01)	–32% (p < 0.01)
Visual scoring (average)	4.5 ± 6	4.6 ± 0.5	3.7 ± 0.8	+1% (p 0.78)	–19% (p < 0.01)	–20% (p < 0.01)

Data presented as mean ± standard deviation (median, minimum-maximum).

In group 2, the calculated contrast dose was below 120 ml in 17 patients and above 120 ml in three patients. Calculated doses higher than 120 ml were due to a combination of high body weight, large stature and/or high heart rate: one patient was given a contrast dose of 126 ml (BW 97 kg, length 175 cm and HR 69 bpm), one patient received a contrast dose of 134 ml (BW 100 kg, length 182 cm and HR 65 bpm) and for one patient a dose of 163 ml was pre-calculated (BW 115 kg, length 185 cm and HR 122 bpm) but this was reduced to the maximum dose of 150 ml per protocol.

**Figure 1:**
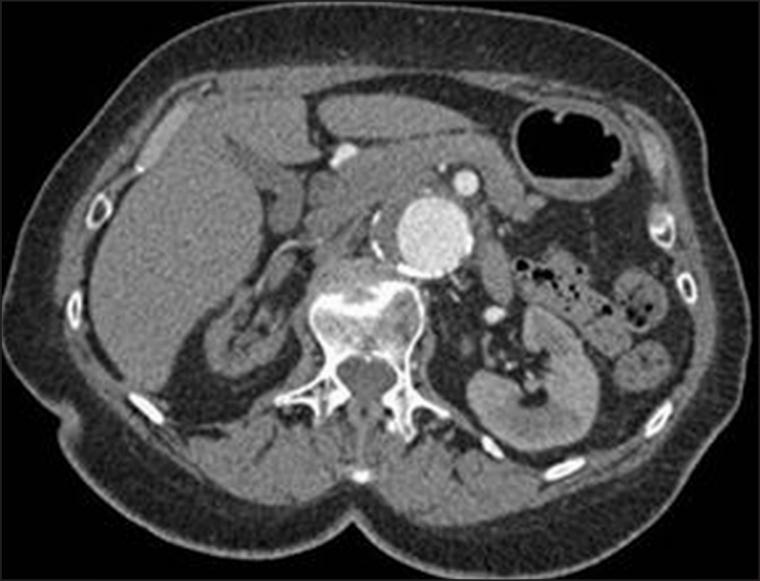
Patient received a contrast dose of 29 ml. Average aortic enhancement was 199.9 HU with visual score of 4/5.

**Figure 2:**
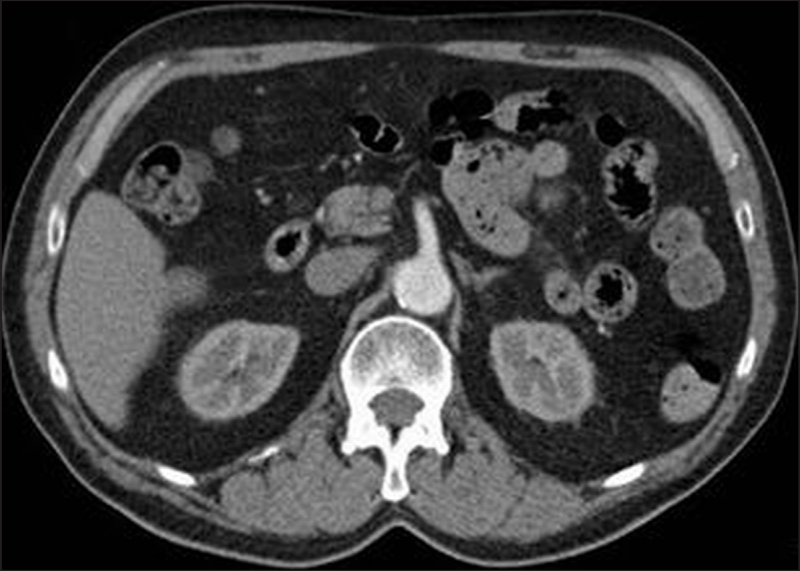
Patient also received a contrast dose of 29 ml. Average aortic enhancement was 188.3 HU with visual score of 4/5.

The lowest contrast dose in this study was 29 ml injected in two patients with an abdominal aortic aneurysm (group 3), both with enhancement near to 200 HU and a visual score of 4/5 (Figures 1 and 2).

### Quantitative image analysis

Average enhancement for the three groups is summarized in Table 3.

### Qualitative image analysis

Subjective image quality scores are summarized in Table 3.

**Figure 3:**
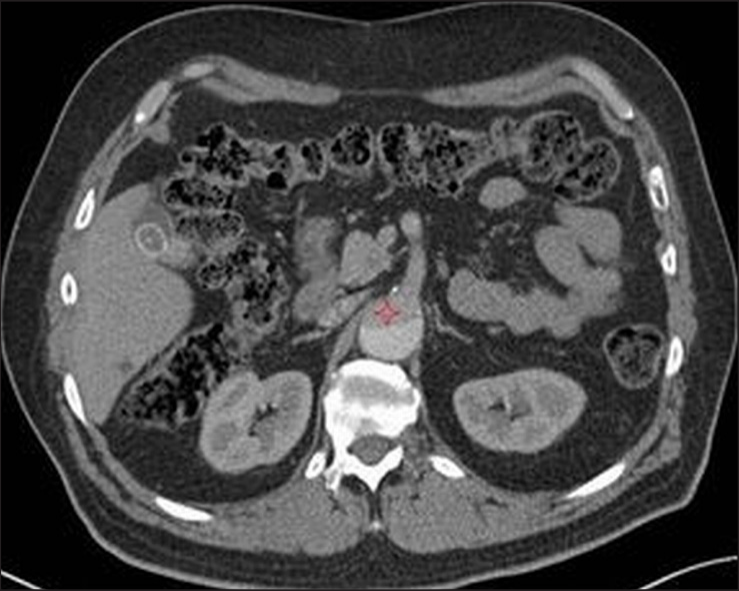
Patient received a contrast dose of 42 ml. Aortic enhancement was 90.5 HU with visual score of 2/5. The red star is placed in the true lumen of the dissection.

**Figure 4:**
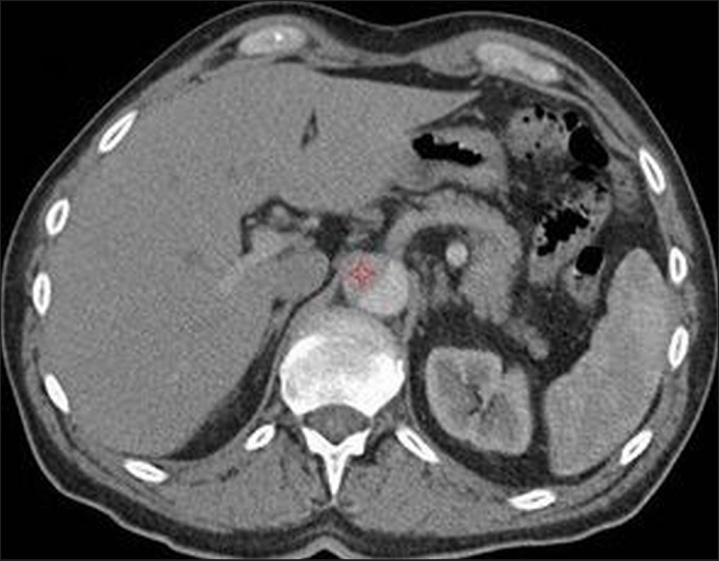
Patient received a contrast dose of 46 ml. Aortic enhancement was 85.5 HU with visual score of 2/5. The red star is placed in the true lumen of the dissection.

Average image quality for group 3 was scored 3.7 with a score of two (suboptimal) in two patients and a score of three in five patients. Two patients with average enhancement below 150 HU still received a score of four, and three patients with a score of three had an average enhancement above 150 HU, of whom two patients above 200 HU (Figures 3 and 4).

Using the intraclass correlation coefficient, applying average measures and consistency, Kappa-values for inter-observer agreement were moderate (0.472), with a consistent difference in scoring: a score of four (good) with one reader versus five (excellent) for the other reader. Inter-observer variability showed a slightly higher average score for group 1 and 2 (+0.4–0.45) and a moderately higher score (+1.1) for group 3 with the second reader, with only one patient of three and no patients with a score of one or two for the second reader. No patients in any of the three study groups received a score of one by any of the readers, and no patients needed to be recalled for repeat imaging.

Intra-observer agreement for the first reader performing a second reading more than one month later, is higher (0.601). Most of the scoring went up at the second scoring time, and the number of scans scored as suboptimal (two patients) remained unchanged.

### Radiation dose

Data on radiation dose is summarized in Table 4. There was no statistical difference between the groups in terms of kV, tube load per rotation, CDTI and DLP. Automatic kV modulation yielded a range of [80–140 kV].

**Table 4:** Radiation dose comparison.

	Group 1	Group 2	Group 3	P Value		

				Group 1 vs 2	Group 1vs 3	Group 2 vs 3

Tube voltage (kV)	115 ± 15.7	119 ±17.7	115 ± 18.2	0.46	0.49	1.00
Exposure time product (mAs)	252.8 ± 78.3	254.8 ± 63.2	258.5 ± 54.5	0.93	0.85	0.79
CDTI vol (mGy)	15.4 ± 6.2	16.5 ± 4.8	15.8 ± 6.6	0.54	0.70	0.85
DLP (mGy*cm)	963 ± 422.8	1089 ± 359.0	1022 ± 501.3	0.32	0.63	0.69

Data presented as mean ± standard deviation.

## Discussion

This study demonstrates that contrast dose could be lowered without lowering enhancement or impairing diagnostic image quality when using an injection algorithm based on BMI and heart rate and implemented on a commercially available contrast agent injector. Also, the range of contrast enhancement can be lowered; however, only the upper limit is affected, while the lower limit remains almost equal. Fewer patients have an unnecessarily high enhancement, while no patients have insufficient contrast enhancement using the proposed injection algorithm.

Reducing the contrast dose with an additional 50% contrast dilution resulted in lower enhancement and diagnostic image quality with an enhancement below 100 HU in three patients and suboptimal image quality (score two) in two patients.

Interestingly, all patients with suboptimal quality and enhancement (below 100 HU) in this study were patients with aortic dissection. This might be related to the unpredictable and sometimes significant redistribution of flow towards the false lumen resulting in too low contrast enhancement within the true lumen, where the contrast opacification was measured. Potentially, patients with large, abdominal aortic aneurysms, associated with a low circumferential thrombus burden, might also have a low or inhomogeneous, intraluminal contrast enhancement, related to the high blood flow turbulence and the low laminar blood flow into the aneurysmal sac.

Adversely, it should be noted that none of the patients in this study needed a repeat scan for insufficient reading quality.

For adjusting contrast dose for CT-angiography the used variables in this study (patient weight, length, heart rate and contrast medium concentration) are not the only parameters to examine; potentially also scanning parameters (such as tube voltage) might influence the impact of contrast agent dose on image quality and diagnostic performance.

There were some limitations to this study. First, patients with moderate renal impairment were preliminary randomly assigned to group 2 or 3 to receive a reduced contrast dose avoiding group 1 with 120 ml of injected contrast. We also compared our calculated doses to a standard contrast dose of 120 ml, which has been historically used for CT-aortography in many institutions. Recent literature, however, suggests that this value is obsolete, and that good quality images can be obtained with significantly lower contrast volumes, using tube voltage modulation [16,17], which can be considered as an alternative technical method to reduce contrast volume. Another limitation is the fact that we arbitrarily set the contrast volume per BSA to a fixed value of 45 ml/m², so far irrespective of any particular characteristics of the contrast agent or the scan parameters. The effects of cardiac output (and heart rate) on CT-image quality are still rather unclear. Therefore we only included patients with a normal heart function; the presented data cannot be extrapolated to patients with cardiac failure.

Finally, we only included patients with screening CT-angiography for aortic aneurysm and dissection. However, in daily practice, other indications for CT-angiography of the abdominal aorta, like follow-up CT after EVAR, were not included in this study.

In conclusion, contrast doses in CT-aortography can be significantly reduced without compromising diagnostic performance, using a contrast injection algorithm based on heart rate and body surface area. Care should be taken in cases suspicious for aortic dissection.
